# Dereplication-Guided Isolation of 3‑*O*‑Methylfunicone from Endophytic Fungal Co-culture and Its Inhibitory Effect on *Phytophthora palmivora*

**DOI:** 10.1021/acsomega.5c08920

**Published:** 2025-11-14

**Authors:** Stéfane M. Q. Santos, Marcus V. A. Marques, Cecília L. S. Pereira, Henrique B. da Silva, Vinicius Palaretti, Viviani N. Takahashi, Sônia C. Oliveira Melo, Eliane O. Silva

**Affiliations:** † Department of Organic Chemistry, Institute of Chemistry, Universidade Federal da Bahia, Salvador, Bahia 40170-115, Brazil; ‡ Department of Biological Sciences, Universidade Estadual de Santa Cruz, Ilhéus, Bahia 45662900, Brazil; § Department of Chemistry, 124588 Faculdade de Filosofia, Ciências e Letras de Ribeirão Preto, Ribeirão Preto, São Paulo 14040900, Brazil

## Abstract

*Phytophthora palmivora* is a destructive pathogen infecting more than 200 plant species, including cocoa, coconut, and papaya. Endophytic fungal co-cultures provide a promising strategy to activate silent biosynthetic pathways and generate bioactive metabolites for crop protection, as endophytes naturally compete for limited resources through the production of specialized metabolites. In this study, we examined the co-culture of *Aspergillus pseudonomiae* J1 and *Talaromyces pinophilus* J6, isolated as endophytes from *Euphorbia umbellata* leaves. Ultrahigh-performance liquid chromatography–high-resolution mass spectrometry (UHPLC–HRMS) data analysis, processed with MS-DIAL and supported by SIRIUS 5 and manual fragmentation proposition, enabled the annotation of quinone, phenylpropanoid, iridoid, and funicone-type natural products. Guided by this analysis, 3-*O*-methylfunicone was isolated, and its chemical structure was confirmed by Nuclear Magnetic Resonance (NMR) and High-Resolution Mass Spectrometry (HRMS) data analysis. Bioassays demonstrated its antifungal activity against *P. palmivora*, inhibiting mycelial growth by 54.9 ± 6.1, 30.9 ± 5.1, and 15.6 ± 6.2% at concentrations of 1.0, 0.5, and 0.25 mM, respectively. Our findings show that metabolomics combined with dereplication offers a powerful strategy to optimize the discovery of bioactive metabolites from endophyte co-cultures.

## Graphical abstract

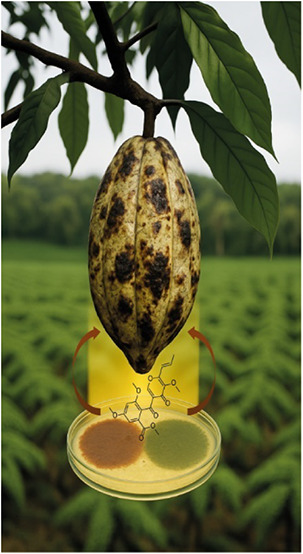

## 1 Introduction

*Phytophthora palmivora* is a hemibiotrophic oomycete with a remarkably broad host range, including economically important food crops such as cocoa (*Theobroma cacao*), coconut (*Cocos nucifera*), and papaya (*Carica papaya*).^1^ In cacao, it causes black pod rot and stem canker, leading to severe yield losses with economic impacts on farmers, agro-industries, and global supply chains.^2^ Management relies on cultivation practices, such as removal of infected pods, combined with fungicide applications (chlorothalonil, mancozeb, mefenoxam, fosetyl-Al).^3^ However, the pathogen’s aggressive infection cycle and its environmental persistence,^4^ along with drawbacks of fungicide use^5^ emphasize the need for more sustainable and effective strategies integrating improved practices, resistant cultivars, and biological control.

The natural products structural diversity, evolved through complex ecological interactions, often confers potent and selective bioactivity against agricultural pests, while reducing unintended impacts on nontarget organisms.^6^ Among their most promising sources are endophytic fungi, which inhabit plant tissues without causing disease and often produce metabolites that enhance host defense against pathogens, pests, and environmental stress.^7^ The often-hostile environments in which endophytic microorganisms live have shaped their ability to survive and adapt, particularly through the biosynthesis of natural products. In natural ecosystems, fungi compete with other microrganisms for limited resources, and one of their primary strategies for survival involves the activation of biosynthetic gene clusters, which leads to the production of specialized metabolites.^8^ These compounds can serve as direct crop protection agents or as scaffolds for the synthesis of novel agrochemicals with improved efficacy and safety profiles.

Under standard laboratory conditions, fungi generally produce only a limited subset of their specialized metabolites repertoire, as many biosynthetic gene clusters remain silent.^9^ As a result, co-cultivation strategies—where fungi are grown in the presence of competing microorganisms—have emerged as a promising approach to trigger these cryptic pathways.^10^ By mimicking natural competitive environments, co-culture can stimulate the production of previously unexpressed metabolites, thereby enhancing chemical diversity and expanding the discovery of novel bioactive compounds.^11^ This approach has proven to be a valuable tool for uncovering novel bioactive compounds, reinforcing its relevance for new chemical discovery.^12,13^

Several studies have shown that co-cultures of endophytic fungi can stimulate the production of natural products active against pathogenic microorganisms.^11^ For example, the biosynthesis of the antifungal stemphyperylenol by *Alternaria tenuissima* markedly increased in co-culture with *Nigrospora sphaerica*.^14^ Similarly, the co-culture of *Nigrospora oryzae* and *Beauveria bassiana* yielded antifungal azaphilones.^15^ On the other hand, the discovery of novel and bioactive specialized metabolites has become increasingly challenging, underscoring the importance of innovative strategies to drive progress in natural products chemistry. Among these strategies, dereplication methods play a pivotal role by enabling the rapid annotation of known bioactive metabolites on natural extracts.^16^ Within the omics pipeline, the critical analysis of spectral data generated by chromatographic tandem mass spectrometry has significantly advanced the characterization of natural products.^17^

*Euphorbia* spp. are renowned for their chemical diversity, particularly diterpenes with antifungal properties,^18^ making their endophytes valuable sources of structurally diverse and bioactive metabolites. As part of our ongoing search for bioactive compounds from endophytic fungal co-cultures, we investigated the interaction between *Aspergillus pseudonomiae* J1 and *Talaromyces pinophilus* J6, both isolated from *Euphorbia umbellata*. UHPLC–HRMS analysis using MS-DIAL revealed a diverse metabolic profile, including quinone, phenylpropanoid, iridoid, and funicone-type natural products, several of which are associated with antifungal activity. From this profile, 3-*O*-methylfunicone was isolated and fully characterized, and its antifungal activity against *P. palmivora* was confirmed. Our results expand the chemical space accessible through fungal co-culture and highlight its potential as a promising strategy for discovering antiphytopathogenic natural products.

## 2 Experimental Procedure

### 2.1 Endophytic Fungi Isolation and Identification

The endophytic fungi coded as J1 and J6 were isolated from aerial parts of *Euphorbia umbellata* (Pax) Bruyns, as previously reported by our research group.^19,20^ The study was registered in the Brazilian System for the Management of Genetic Heritage and Associated Traditional Knowledge (SisGen) under code AE18457.

Fungal strains were identified by DNA sequencing followed by phylogenetic analysis. Genomic DNA was extracted from 7-day-old cultures using mechanical disruption with glass microspheres (425–600 μm diameter; Sigma-Aldrich), followed by RNase treatment, phenol:chloroform:isoamyl alcohol extraction, and precipitation with isopropanol, as previously described.^21^

PCR amplification was carried out using gene-specific markers: the β-tubulin (benA) locus for strain J6 with primers Bt2a/Bt2b, and the calmodulin (cmdA) locus for strain J1 with primers Cf1/Cf4. PCR reactions were performed in a Thermal Cycler (C1000 Touch) under conditions previously described^22^ and amplification was confirmed by agarose gel electrophoresis and observed under ultraviolet light before purification. Amplicons were then purified using the GFX PCR DNA and Gel Band Purification Kit (GE Healthcare) and sequenced on an ABI 3500XL Genetic Analyzer (Applied Biosystems).

Consensus sequences were manually edited and assembled using BioEdit (ver. 7.2.6), then compared against sequences in the NCBI database with the Basic Local Alignment Search Tool (BLAST; https://blast.ncbi.nlm.nih.gov/). Fungal identification was assigned based on the highest sequence similarity with reference strains deposited in GenBank.

For phylogenetic analysis, sequences were aligned with ClustalX^23^ and analyzed in MEGA (ver. 11.0).^24^ Phylogenetic trees were reconstructed using the Neighbor-Joining method with the Kimura two-parameter (K2P) model,^25^ which allowed the calculation of evolutionary distance matrices. Future studies should incorporate multilocus data sets analyzed under Maximum Likelihood approaches to provide a more robust and reliable taxonomic classification.

Branch support was evaluated with 1,000 bootstrap replicates. The obtained sequences were deposited in GenBank under accession numbers MW600554 (strain J1) and PQ963936 (strain J6). Both isolates are preserved in the culture collection of our laboratory.

### 2.2 Endophytic Fungi Culture and Extraction

The endophytic fungi were cultivated in dual (co-culture) and single (axenic) cultures in Petri dishes containing 20 mL of potato dextrose agar (PDA, Kasvi). Single cultures were established by adding plugs (6 mm diameter) from the fungus, while for the dual cultures, plugs from two different fungi (a plug from each) separated by 5 cm were placed simultaneously in the culture medium. All cultures were carried out in triplicate and incubated in a BOD chamber for 10 days, at 28 °C. After incubation, the entire culture (including both the mycelium and the culture medium) was extracted by adding 40 mL of ethyl acetate. The mixture was sonicated for 20 min to enhance metabolite release, and the organic solvent was then evaporated under reduced pressure to yield the crude extract. The blank was achieved under the same conditions and consisted of the PDA without fungus.

### 2.3 UHPLC–HRMS Analysis

All crude extracts from single and dual endophyte cultures were analyzed by UHPLC–HRMS. UHPLC–HRMS apparatus (Thermo Fisher Scientific) contained an electrospray ionization (ESI) source and an Orbitrap analyzer. The flow rate was 400 μL/min, and the gradient elution system was 5 to 100% methanol (HPLC grade, Tedia, Rio de Janeiro, Brazil) in water for over 30 min. A C18 column (ACE 150 mm × 4.6 mm × 3 μm) was used at a spectrometer operating at both positive and negative modes. The column temperature was set at 30 °C. The following parameters were used: scanning range of 120–1200 *m*/*z* to full MS, ESI MS resolution of 70,000 with lock mass, microbeam of 1, and maximum injection time of 250 ms. The parameters of the ESI ionization source were as follows: gas flow rate of 30 L/min; auxiliary gas flow rate of 10 L/min; positive voltage spray mode of 3.6 kV; negative voltage spray mode of 3.2 kV; and S-lens level of 55. Nitrogen gas was used as a nebulizer in the collision cell. The mass spectra were obtained and processed using XCalibur software (Thermo Fisher Scientific).

### 2.4 Metabolomics and Dereplication

Each culture replicate was extracted and analyzed independently by UHPLC–HRMS to capture biological variability. The resulting raw data (positive ion mode) were processed using MS-DIAL software (ver. 4.9), and the features detected across the triplicates were aligned and compared to ensure reproducibility.

Data collection was performed with 0.01 Da MS1 tolerance and 0.05 Da MS2. Peak detection was applied with 5E5 amplitude for minimum peak height (threshold) and 0.1 Da mass slice width. Deconvolution parameters were set as follows: sigma window value of 0.5 and MS2 abundance cutoff of 10 amplitudes. Alignment parameters setting included as reference file the QC sample (most complex file), a retention time tolerance of 0.05 min. Finally, features based on blank information were removed. The peak area and peak height for each detected peak, representing the abundance of a compound in the sample, were calculated.

Following data processing and feature extraction, a dereplication workflow was applied as part of the metabolic profiling analysis. Molecular formulas were generated using XCalibur software, applying the nitrogen rule and maximum mass error of 5 ppm. Putative metabolite candidates were retrieved with the SIRIUS 5 platform and ranked based on similarity scores derived from comparisons between experimental MS2 spectra and reference spectra in the database. The top-ranked candidates were assigned, and key fragment ions were analyzed to support the most plausible structural proposals.

### 2.5 Isolation and Structural Identification of the Main Specialized Metabolite

The co-culture was carried out in 100 Petri dishes containing PDA medium, incubated in a BOD chamber for 10 days, at 28 °C. The specialized metabolites were extracted using ethyl acetate. The achieved extract was dried over anhydrous sodium sulfate and concentrated under vacuum to yield the crude extract.

The purification process was carried out on a chromatographic column (40 × 1.5 cm) containing silica gel 60 A (Sigma-Aldrich). The mobile phase consisted of gradients composed of hexane (Synth, São Paulo, Brazil), ethyl acetate (Synth, São Paulo, Brazil), and methanol (Synth, São Paulo, Brazil).

The main specialized metabolite from the co-culture was isolated following a dereplication-guided approach. UHPLC–HRMS analysis of the co-culture and monocultures identified unique peaks present only in the co-culture (or in higher amounts), indicating metabolites specifically induced by fungal interaction. Among these, the peak corresponding to 3-*O*-methylfunicone was prioritized for isolation based on its abundance and potential bioactivity suggested in previous studies.

One and two-dimensional-NMR spectra were recorded at 500 and 125 MHz for ^1^H and ^13^C, respectively, with a DRX 500 spectrometer (Bruker, Billerica, USA). Chemical shifts (δ) were referenced to the residual deuterated methanol (CD_3_OD) peak at δH 3.31 for ^1^H and δC 49.00 for ^13^C.

(*E*)-3-Methoxy-2-propenyl-5-(2′-carbomethoxy-4′-6′-dimethoxybenzoyl)-4-pyrone or 3-*O*-methylfunicone: white powder; ^1^H NMR (500 MHz, CD_3_OD): δ 8.48 (1H, *s*, H-6); 7.07 (1H, *d*, *J* = 2.0 Hz, H-3′); 6.81 (1H, *d*, *J* = 2.0 Hz, H-5′); 6.74 (1H, *m*, H-8); 6.61 (1H, *dd*, *J* = 3.1, 7.3, H-7), 3.88 (3H, *s*, H-10’), 3.78 (3H, *s*, H-10), 3.76 (3H, *s*, H-8’), 3.75 (3H, *s*, H-9’), 1.97 (3H, *dd*, *J* = 1.5 and 7.0 Hz, H-9); ^13^C NMR (125 MHz, CD_3_OD): δ 192.8 (C-11), 174.3 (C-4), 168.0 (C-7’), 163.1 (C-4’), 161.5 (C-6), 159.5 (C-6’), 156.8 (C-2), 145.2 (C-3), 137.4 (C-8), 132.0 (C-2’), 128.0 (C-5), 126.0 (C-1’), 119.3 (C-7), 107.3 (C-3′), 103.6 (C-5′), 61.1 (C-10), 56.7 (C-9’), 56.3 (C-10’), 52.9 (C-8’), 19.0 (C-9). HR-MS: 389.1244 ([M + H]^+^; (C_20_H_20_O_8_)­H^+^; calc. 389.1231; error 3.3 ppm).

### 2.6 Antifungal Assay against the Phytopathogenic *Phytophthora palmivora*

The plant pathogenic fungal strain *Phytophthora palmivora* MCCS-MB-01919 was obtained from the multinational company MCCS—Mars Center for Cocoa Science (Brazil). The fungus was maintained on carrot agar medium (CAM) plates at 28 °C and subcultured every 7 days to ensure viability. For the assay, fresh cultures (7 days old) were used to prepare the inoculum.

The antifungal activity of the different treatments was determined using the agar dilution method to determine the inhibitory effect of the 3-*O*-methylfunicone on the mycelial radial growth of the *P. palmivora*, according to EUCAST standard antifungal susceptibility testing procedures.^26^

The evaluated compound (3-*O*-methylfunicone) was dissolved in dimethyl sulfoxide (DMSO) to prepare the working solutions at three concentrations (1.0, 0.5, and 0.25 mM). For the assays, aliquots of 0.2 mL of the compound solutions were evenly spread onto the surface of the culture medium.

As controls, 0.2 mL of distilled water (zero control) and 0.2 mL of 3% DMSO (negative control) were applied. Additionally, quality control (QC) assays were performed in accordance with EUCAST guidelines. Routinely, QC strains were included to ensure the accuracy and reliability of the assays.

Subsequently, one mycelial plug (⌀ = 8 mm) of a 7-days culture of *P. palmivora* was placed at the center of each plate containing the above-mentioned concentrations for each treatment. The plates were incubated at 25 °C under continuous illumination provided by a fluorescent lamp. Measurements (radial mycelial growth in mm) were taken when the mycelium of the zero control had completely covered the surface of the plate.

Based on the obtained values, the percentage of mycelial growth inhibition (MGI) was calculated using the following equation:^27^

$$\mathrm{MGI}=\left[\frac{\mathrm{MC}-\mathrm{MT}}{\mathrm{MC}}\right]\times 100$$

where MC represents the diameter of the fungal colony of the control group and MT represents the mycelial growth diameter in the treatment group.

All assays were performed in triplicate. Data were expressed as mean ± standard deviation (SD).

## 3 Results and Discussion

### 3.1 Endophytic Fungi: Co-culture and Molecular Identification

Two fungal strains were isolated as endophytes from the aerial parts of *Euphorbia umbellata* and designated as J1 and J6. These strains were cultivated individually and in dual culture on Petri dishes containing PDA for 10 days. In a previous study, we evaluated all possible combinations among all isolated endophytic fungi from *E.umbellata* to assess their individual chemical responses under confrontation conditions.^28^ Using an untargeted metabolomics approach, that study revealed that all co-cultures involving the J6 strain exhibited distinct chemical profiles, with the J1-J6 combination standing out due to its unique metabolomic fingerprint. These findings highlighted the biosynthetic potential of J6 when challenged with other microorganisms, prompting a deeper investigation into its interaction with J1.

The J1 and J6 strains (macroscopic morphologies are shown in Figure S1 in the Supporting Information) were identified using molecular techniques. Phylogenetic trees constructed based on the calmodulin gene for J1 and the β-tubulin gene for J6, together with sequences from closely related species, are presented in Figure S2 (Supporting Information). Accurate identification of endophytic fungi requires molecular analysis involving DNA sequencing and phylogenetic inference using specific genetic markers. Among these, protein-coding genes such as actin (ACT), translation elongation factor 1α (TEF-1α), β-tubulin, and calmodulin have been widely recommended due to their high resolution in distinguishing closely related or cryptic species, as well as for elucidating phylogenetic relationships among different fungal species within the same genus.^29^

Analysis of the calmodulin nucleotide sequence of strain J1 revealed that it displays high sequence similarity (96%) with *Aspergillus pseudonomiae* Varga, Samson and Frisvad 2011.^30^ *Aspergillus* section *Flavi* comprises 22 species that are well-known for their ability to produce aflatoxins.^30,31^ In a previous study, our research group reported the antiprotozoal activity of three lactones isolated from single cultures of *A. pseudonomiae* J1 against *Trypanosoma cruzi*. Notably, (+)-phomolactone was more potent than benznidazole to inhibit both epimastigotes and trypomastigotes forms.^19^

The alignment of the β-tubulin sequence of J6 and the type strains of *Talaromyces pinophilus* CBS 631.66 (JX091381) gave an identity of 99%. The genus *Talaromyces* is widely distributed across diverse environments, including soil, plant tissues, and marine habitats. Scientific interest in metabolites produced by *Talaromyces* species (phylum Ascomycota) has grown steadily, as these fungi are recognized as valuable sources of natural products. They are capable of producing a wide array of structurally diverse specialized metabolites with notable biological activities, making them attractive targets for bioprospecting efforts.^32^

### 3.2 Dereplication of Extract from Co-culture between *Aspergillus pseudonomiae* J1 and *Talaromyces pinophilus* J6

Current dereplication workflows have significantly advanced the characterization of natural products, driven by the expansion of comprehensive natural product databases, substantial improvements in analytical technologies for chemical profiling, and the rapid development of artificial intelligence tools.^33^ Dereplication of metabolites produced in the co-culture between *A. pseudonomiae* J1 and *T. pinophilus* J6 was performed using LC-MS/MS data combined with computational analysis via MS-DIAL and SIRIUS platforms.

The metabolomic profile of the co-culture revealed a set of features with abundances differing from those in the respective monocultures, supporting the hypothesis that microbial interactions can activate or suppress biosynthetic gene clusters. In total, MS-DIAL analysis identified 315 compounds in the co-culture. The complete list of detected features, including retention time, *m*/*z*, and peak area for each condition, is provided in Table S1 (Supporting Information).

Detailed analysis of the base peak chromatograms (BPC) of the J1–J6 dual culture extract showed the majority of peaks between 14 and 26 min of retention time (Figure 1). Comparison of the BPCs (Figure 1A–C) highlighted prominent peaks at 14.82, 15.87, 17.77, 23.44, and 24.08 min, corresponding to compounds **1–5**, whose production levels were altered upon coculture establishment.

**Figure 1:**
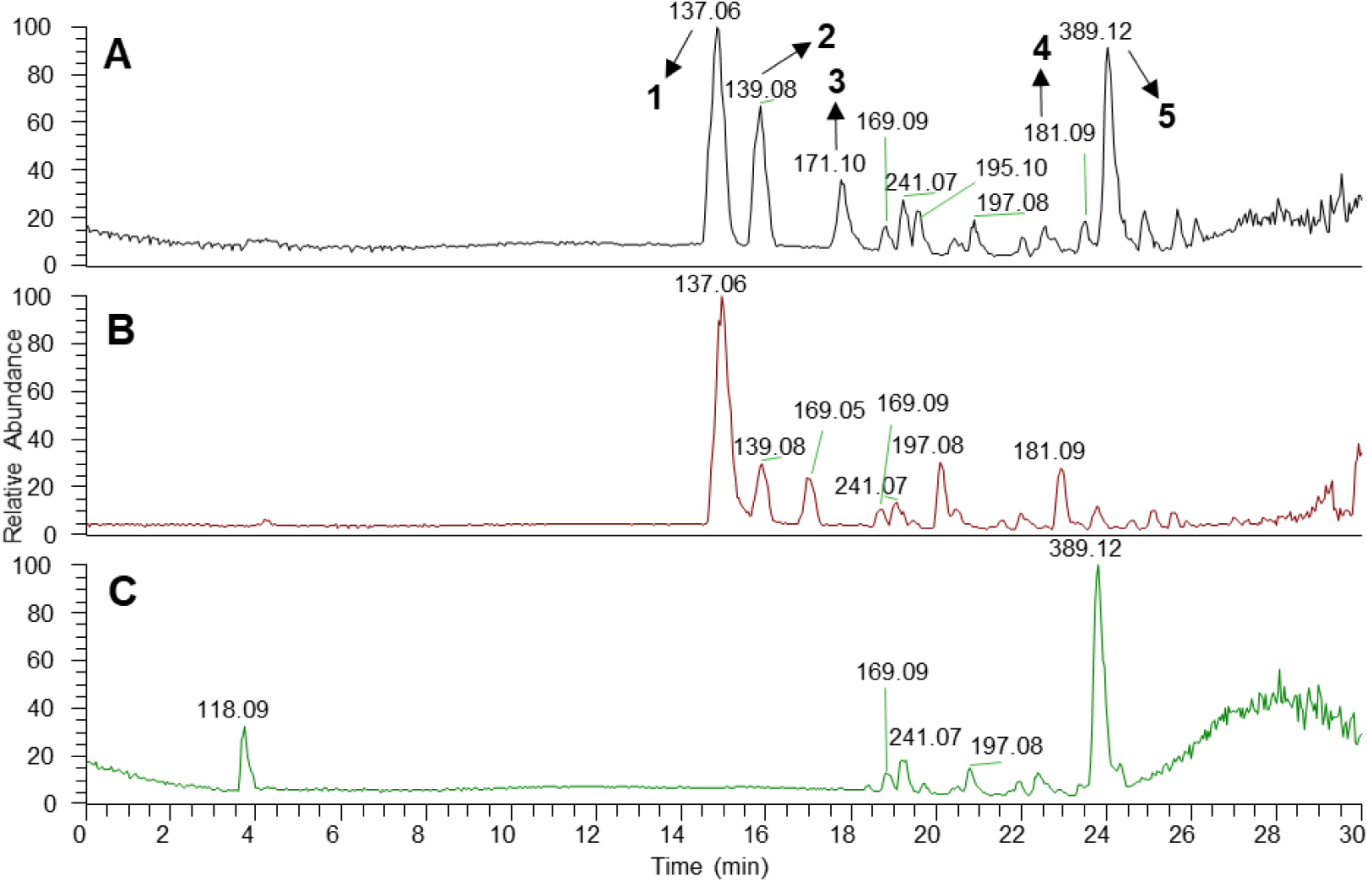
Base peak chromatograms (BPC) from UHPLC–HRMS of the ethyl acetate extract of *Aspergillus pseudonomiae* J1–*Talaromyces pinophilus* J6 dual culture (A), *Aspergillus pseudonomiae* J1 (B), and *Talaromyces pinophilus* J6 (C). Compounds **1**–**5** were correlated with the dual culture establishment and were putatively identified. Compound **5** was additionally isolated and identified by NMR data analysis.

The relative abundances of compounds **1–5** in single and dual cultures were evaluated using MS-DIAL (Figure 2). Compounds **1**, **2**, and **4** appear to be primarily associated with *A. pseudonomiae* J1 metabolism, whereas compound **5** is mainly produced by *T. pinophilus* J6. Compound **3** was almost exclusively detected in the co-culture. Comparative analysis of the co-culture extract revealed a shift in metabolite production: the level of compound **2** increased, while compounds **1**, **4**, and **5** decreased. These observations indicate that the interaction between the two fungi selectively modulated the biosynthesis of these specific metabolites, highlighting the dynamic metabolic interplay in co-culture conditions.

**Figure 2:**
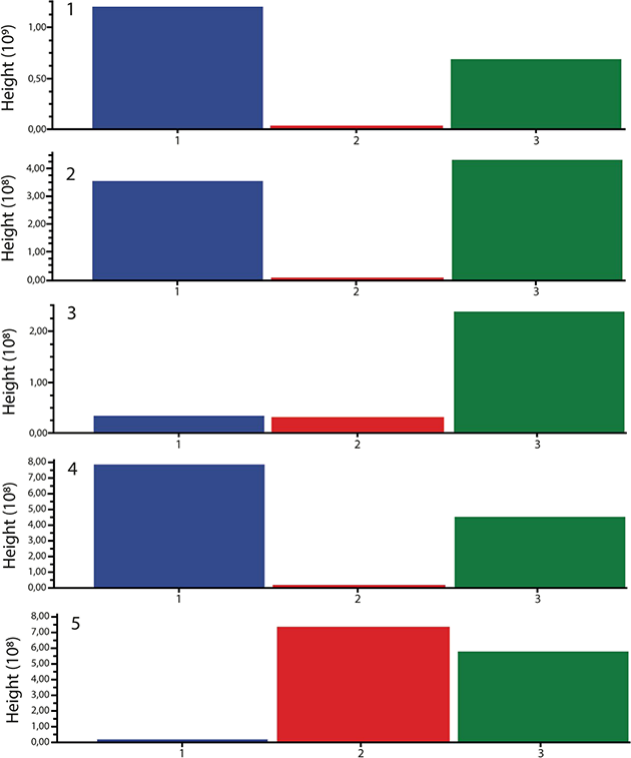
Relative amounts of annotated compounds **1**–**5** in *Aspergillus pseudonomiae* J1 single culture (blue bar), *Talaromyces pinophilus* J6 single culture (red bar), and *Aspergillus pseudonomiae* J1–*Talaromyces pinophilus* J6 dual culture (green bar).

With advances in metabolomics, genomics, and fermentation technology, the exploration of endophytic fungi is increasingly recognized as a sustainable and innovative approach for sourcing bioactive natural products. Recent research on fungal co-cultivation has confirmed its potential to activate dormant biosynthetic gene clusters, significantly expanding the chemical diversity of natural products. Our findings demonstrate the effectiveness of combining computational dereplication workflows with co-culture-based approaches to expand the accessibility of promising bioactive compounds from microbial sources.

To comply with established metabolomics standards, putative metabolite **1**–**5** annotations were assigned at confidence level 2 according to the Metabolomics Standards Initiative.^34^ For each metabolite feature, the SIRIUS 5 platform generated a ranked list of candidate structures by comparing experimental MS2 spectra with *in silico* fragmentation patterns. Similarity scores analysis and manual curation based on fragment assignments were performed to determine the most plausible molecular identity. Final candidates were cross-referenced against the SciFinder database. Five metabolites (**1**–**5**), whose biosynthesis were associated with the co-culture, were then putatively identified (Table 1).

**Table 1:** Annotated Specialized Metabolites in the *Aspergillus Pseudonomiae* J1–*Talaromyces Pinophilus* J6 Co-culture Using UHPLC-ESI-QTOF-MS and Dereplication^a^

No	RT (min)	Theoretical/observed mass (error, ppm)	Putative identification, adduct	Molecular formula	J1 area	J6 area	Co-culture area
1	14.82	137.0597/137.0604 (5.11)	phlorone, [M + H]^+^	C_8_H_8_O_2_	3.595 x 10^10^	1.064 x 10^9^	2.124 x 10^10^
2	15.87	139.0754/139.0760 (4.32)	4-hydroxy-2,5-dimethylcyclohexa-2,5-dien-1-one, [M + H]^+^	C_8_H_10_O_2_	1.121 x 10^10^	2.413 x 10^8^	1.287 × 10^10^
3	17.77	171.1016/171.1024 (4.67)	isoboonein, [M + H]^+^	C_9_H_14_O_3_	1.132 × 10^9^	1.344 × 10^9^	1.021 × 10^10^
4	23.44	181.0859/181.0868 (4.97)	coniferyl alcohol, [M + H]^+^	C_10_H_12_O_3_	7.084 x 10^9^		2.982 × 10^9^
5	24.08	389.1231/389.1246 (3.85)	3-*O*-methylfunicone, [M + H]^+^	C_20_H_20_O_8_	nd	1.686 × 10^10^	1.304 × 10^10^

a Peak areas of the corresponding compounds in the axenic cultures are included for comparison (nd: not detected).

The MS2 spectra of compounds **1**–**5**, along with their proposed chemical structures and fragmentation pathways, are shown in Figures 3, 4 and 5 (MS1 spectra of 1–5 are shown in Figure S3 in Supporting Information). Manual curation of each MS2 spectra was performed to improve the reliability of compounds annotation. For this purpose, the fragmentation patterns obtained by HRMS were carefully analyzed and proposed.

**Figure 3:**
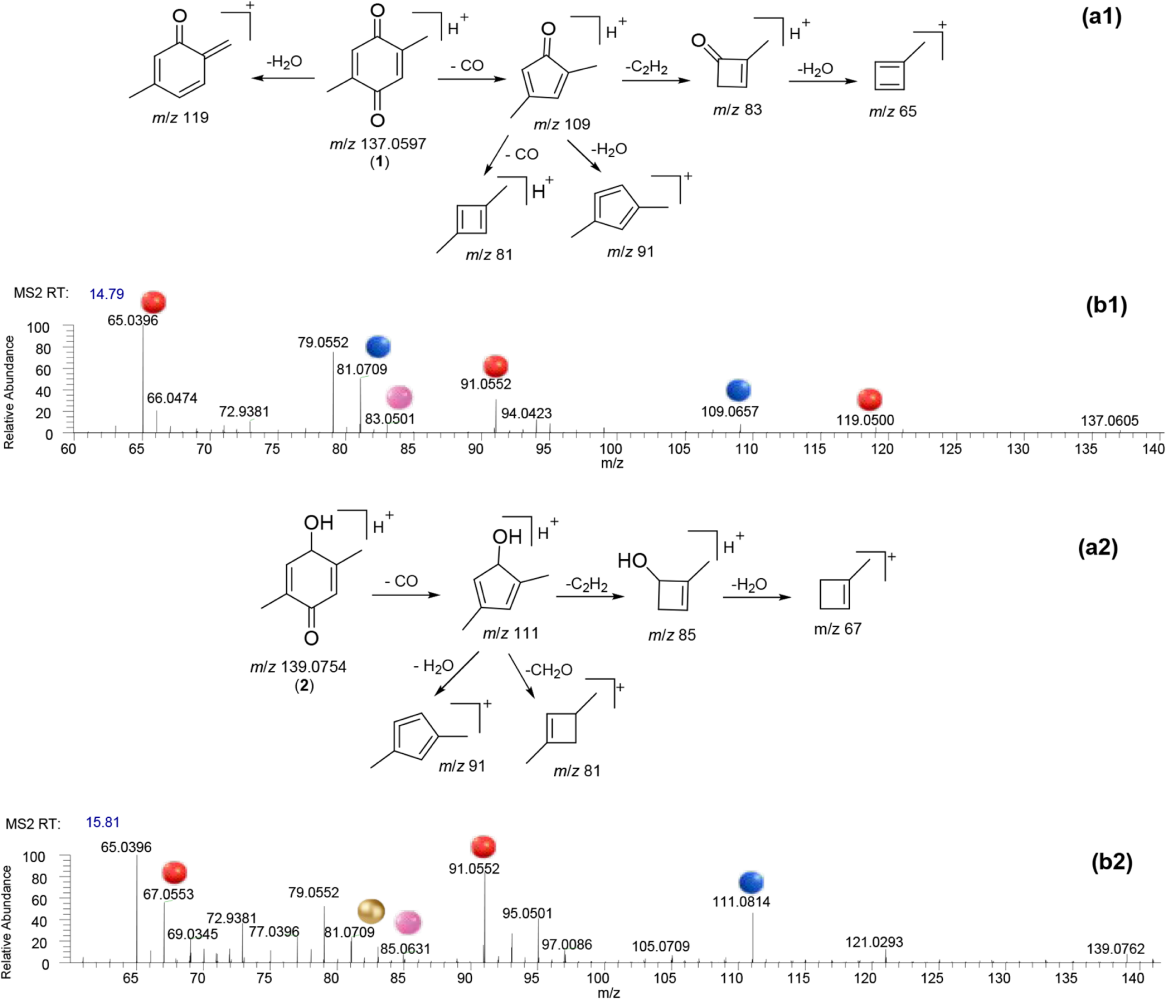
Proposed fragmentation of phlorone (a1) and 4-hydroxy-2,5-dimethylcyclohexa-2,5-dien-1-one (a2) via successive ring contractions. MS2 spectra (positive ion mode) of phlorone and 4-hydroxy-2,5-dimethylcyclohexa-2,5-dien-1-one (b1 and b2, respectively). Ions formed from the loss of H_2_O, CO, C_2_H_2_, and CH_2_O are shown by red, blue, pink, and gold spheres, respectively.

**Figure 4:**
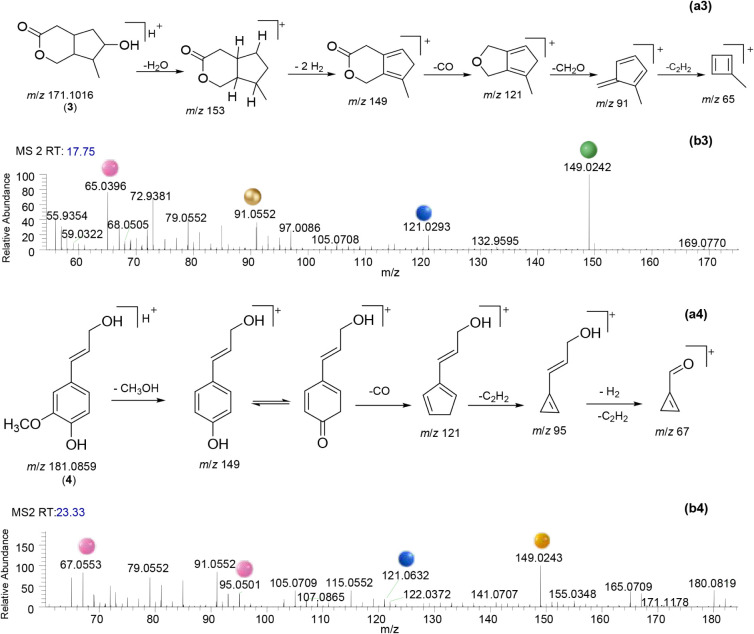
Proposed fragmentation of isoboonein (a3) and coniferyl alcohol (a4) via successive ring contractions. MS2 spectra (positive ion mode) of isoboonein and coniferyl alcohol (b3 and b4, respectively). Ions formed from the loss of CO, C_2_H_2_, H_2_, CH_2_O, and CH_3_OH are shown by blue, pink, green, gold, and yellow spheres, respectively.

**Figure 5:**
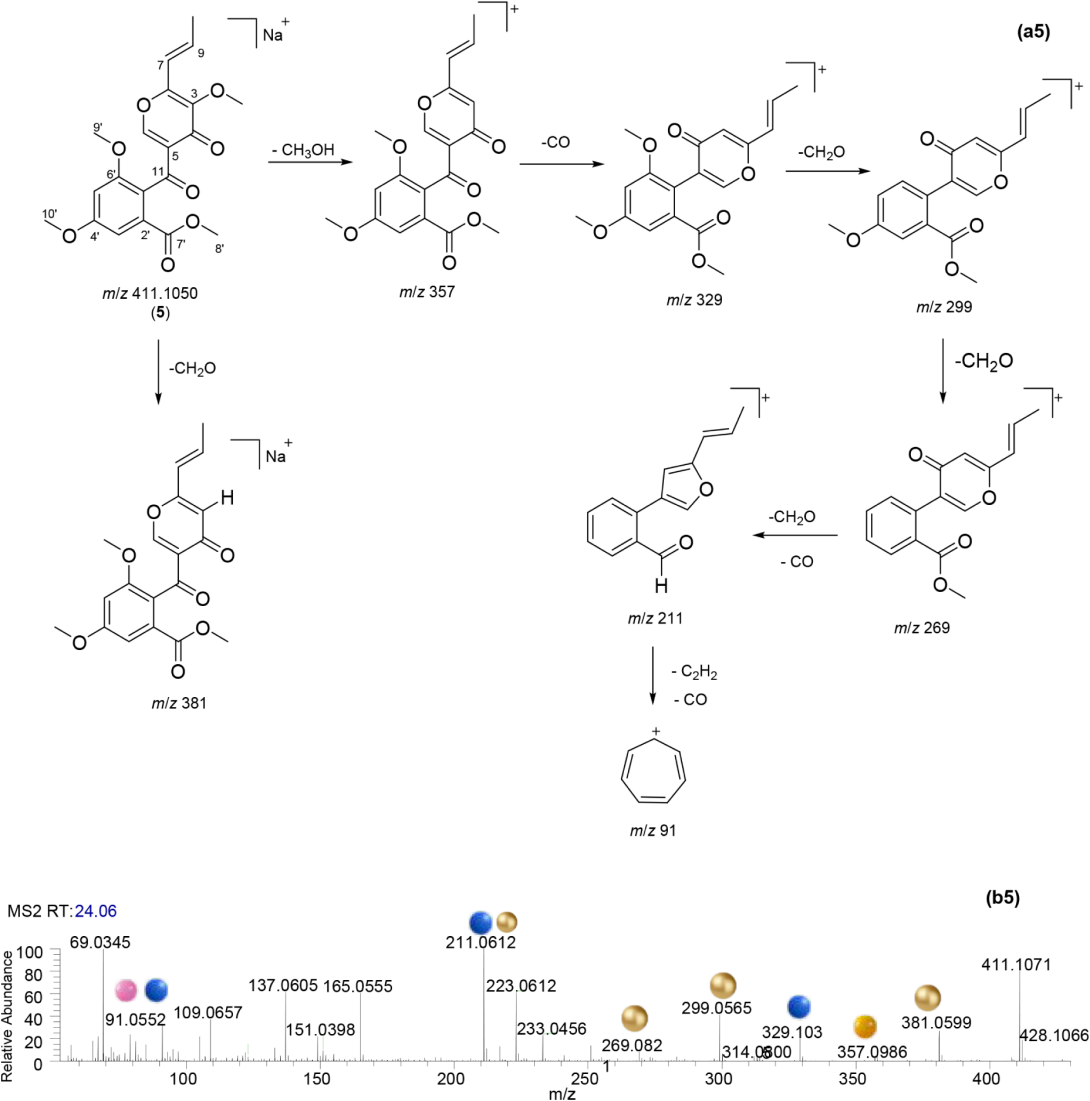
Proposed fragmentation of 3-*O*-methylfunicone (a5) and its MS2 spectra (positive ion mode, b5). Ions formed from the loss of CH_3_OH, CO, CH_2_O, and C_2_H_2_ are shown by yellow, blue, gold, and pink spheres, respectively.

The MS1 spectrum (Figure S3A in Supporting Information) of phlorone (**1**), a quinone, displayed a base peak at *m*/*z* 137.0604 [M + H]^+^, consistent with the molecular formula C_8_H_8_O_2_. This ion was also observed in its MS2 spectrum (Figure 3b1), where it generated a fragment at *m*/*z* 119 by dehydration. Alternatively, CO loss from *m*/*z* 137 produced the ion at *m*/*z* 109 through ring contraction. Successive ring contractions from *m*/*z* 109, through losses of CO, H_2_O, and C_2_H_2_, yielded ions at *m*/*z* 81 (Δ*m*/*z* = 28 Da), 91 (Δ*m*/*z* = 18 Da), and 83 (Δ*m*/*z* = 26 Da), respectively. Finally, the dehydration of ion at *m*/*z* 83 led to the base peak at *m*/*z* 65 (Figure 3a1). Microbial cocultures have revealed the production of quinone derivatives,^35^ compounds widely recognized for their ecological roles as redox-active metabolites that mediate interspecies competition, signaling, and defense mechanisms.

Compound **2** exhibited a base peak at *m*/*z* 139.0760 ([M + H]^+^, C_8_H_10_O_2_) in the MS1 spectrum (Figure S3B in Supporting Information), corresponding to a molecular formula with one degree of unsaturation less than phlorone (**1**). In the MS2 spectrum (Figure 3b2), the precursor ion at *m*/*z* 139 underwent fragmentation via ring contraction. The initial fragmentation involved the neutral loss of CO, yielding a fragment ion at *m*/*z* 111. As observed for **1**, losses of H_2_O, CH_2_O, and CH_2_H_2_ from ion at *m*/*z* 111 produced fragment ions at *m*/*z* 91 (base peak), 81, and 85, respectively. Dehydration of ion at *m*/*z* 85 led to the peak at *m*/*z* 67 (Figure 3a2).

The MS1 spectrum (Figure S3C in Supporting Information) of isoboonein (**3**) displayed a pseudomolecular ion at *m*/*z* 171.1024, consistent with the calculated [M + H]^+^ for the molecular formula C_9_H_14_O_3_. In the MS2 spectrum of **3** (Figure 4b3), the base peak at *m*/*z* 149 was generated by sequential losses of H_2_O and H_2_ from the precursor ion (*m*/*z* 171). Subsequent fragmentation involved sequential neutral losses of CO, CH_2_O, and C_2_H_2_ from *m*/*z* 149, leading to ring contractions that yielded ions at *m*/*z* 121, 91, and 65, respectively. This fragmentation pathway (Figure 4a3) supports the presence of the iridoid scaffold and confirms the proposed structure of isoboonein (**3**).

The MS1 spectrum (Figure S3D in Supporting Information) of coniferyl alcohol (**4**) displayed a pseudomolecular ion at *m*/*z* 181.0868, consistent with the [M + H]^+^ ion for the molecular formula C_10_H_12_O_3_. In the MS2 spectrum (Figure 4b4), the precursor ion (*m*/*z* 181) underwent characteristic fragmentations (Figure 4a4). The primary fragmentation involved the loss of a methanol molecule (Δ*m*/*z* = 32 Da), generating a prominent ion at *m*/*z* 149. Subsequent ring contractions led to the formation of ions at *m*/*z* 121, 95, and 67, corresponding to the sequential losses of CO, and two C_2_H_2_ units. Overall, these fragmentations corroborate the presence of the hydroxycinnamoyl structure and support the proposed structure of coniferyl alcohol (**4**).

Finally, the compound **5** was putatively identified as 3-*O*-methylfunicone, a polyketide widely found in the *Talaromyces* genus. Two peaks were observed at MS1 spectrum (Figure S3E in Supporting Information) of compound **5**, at *m*/*z* 389.1246 and *m*/*z* 411.1065, corresponding to [M + H]^+^ and [M + Na]^+^, respectively. The fragmentation pathway (Figure 5a5) of compound **5** was deduced with basis on its MS2 spectrum (Figure 5b5) and supported its proposed chemical structure. Initial CH_2_O loss from *m*/*z* 411 generated the ion at *m*/*z* 381. On the other hand, neutral CH_3_OH loss from *m*/*z* 411 generated *m*/*z* 357, which was transformed at ions at *m*/*z* 329, 299, 269, 211, and 91 by sequential losses of CO, CH_2_O, and C_2_H_2_.

As previous mentioned, *P. palmivora* is a highly aggressive phytopathogen that severely affects economically important crops, highlighting the urgent need for alternative and environmentally friendly control strategies. In this study, five natural products (compounds **1**–**5**) were putatively identified in the extract of endophyte co-culture, providing a possible link between the chemical diversity induced by microbial interaction and antifungal potential. Quinones such as phlorone (**1**) have been isolated from *Talaromyces* spp.,^36^ but, to the best of our knowledge, no antifungal activity has been described for this compound type. Isoboonein (**3**) has been previously assessed for antimicrobial effects but was only weakly active.^37^ Despite the lack of previously reported antimicrobial activity for compound **3**, its almost exclusive detection in the co-culture prompted us to attempt its isolation. However, the amount obtained was insufficient for NMR characterization or antifungal assays. While its effect on *P. palmivora* remains unknown, we suggest that further studies are needed to evaluate the biological potential of isoboonein (**3**) against *P. palmivora*. On the other hand, coniferyl alcohol (**4**) has been associated with the inhibition of fungal species responsible for Botryosphaeria dieback in grapevine.^38^ Among the metabolites detected, 3-*O*-methylfunicone (**5**), a benzo-γ-pyrone derivative, has attracted particular attention due to its reported antifungal properties. This metabolite has been characterized as fungitoxic, inhibiting the growth of several phytopathogenic fungi, and thus represents a promising candidate for agricultural biocontrol applications.^39^ Given both its reported antifungal potential and its high abundance in the *A. pseudonomiae* J1-*T. pinophilus* J6 co-culture extract (Figure 1 and Table 1), our efforts were directed toward the isolation of 3-*O*-methylfunicone.

### 3.3 Isolation, Identification, and Antifungal Activity of 3-*O*-Methylfunicone from *Aspergillus pseudonomiae* J1–*Talaromyces pinophilus* J6 Dual Culture

*A. pseudonomiae* J1 and *T. pinophilus* J6 were co-cultivated on a large scale in order to isolate the main annotated metabolite (3-*O*-methylfunicone, **5**) and confirm its chemical structure by NMR and HRMS data analysis. The scaled-up extract was subjected to column chromatography, affording a compound with a pseudomolecular ion at *m*/*z* 389.1244 in the HRMS spectrum (Figure S8 in Supporting Information), consistent with the molecular formula C_20_H_20_O_8_. The 1D and 2D NMR spectroscopic data (Figures S4–S7 in Supporting Information) allowed its unequivocal structural identification as 3-*O*-methylfunicone.^39^ Briefly, the singlets at δ 3.88, 3.78, 3.76, and 3.75 were attributed, respectively, to methoxyl groups at positions 10’, 10, 8’, and 9’. The two ethylenic hydrogens (δ 6.61 and 6.74, at C-7 and C-8, respectively) were in *E* configuration because of the mutual coupling constant (*J* = 15.0 Hz). Moreover, the methyl hydrogens at δ 1.97 coupled with C-7 (δ 119.3) and C-8 (δ 137.4), which allowed us to suggest a propenyl side chain. The aromatic H-3′ and H-5′ (δ 7.07 and 6.81) had a meta coupling constant (J = 2.0 Hz). Signals at δ 174.3, 161.5, 156.8, 145.2, and 128.0 in ^13^C NMR, along with the 2D correlations with methine hydrogen δ 8.48, were in good agreement for a γ-pyrone nucleus.

Therefore, initially, the metabolite **5** (Table 1) at retention time 24.08 min was putatively identified as 3-*O*-methylfunicone, and now its identification has been confirmed by NMR data analysis. It is worth mentioning that the isolation and structural identification of 3-*O*-methylfunicone validated our dereplication approach.

Funicone-like compounds are characterized by a γ-pyrone ring linked to an α-resorcylic acid nucleus through a ketone. They have frequently been isolated from *Talaromyces* genus and display a range of biological activities.^40^ Funicones are fungal polyketides that have been characterized as determinants of the antagonistic abilities by the producers against other microorganisms.^41^

Prompted by the previously reported antifungal potential of 3-*O*-methylfunicone, its inhibitory activity was evaluated against the phytopathogenic fungus *P. palmivora*. The compound displayed a significant inhibitory effect, as evidenced by a pronounced reduction in the mycelial growth of *P. palmivora* compared with the control. The percentage of mycelial growth inhibition reached 54.9 ± 6.1, 30.9 ± 5.1, and 15.6 ± 6.2% at concentrations of 1.0, 0.5, and 0.25 mM of 3-*O*-methylfunicone, respectively. These results demonstrate that the evaluated polyketide possesses noteworthy antifungal potential and may represent a promising candidate for the biological management of crop diseases caused by *P. palmivora*.

Polyketides represent an important class of bioactive natural products with a broad range of biological activities. The inhibition of *P. palmivora* by 3-*O*-methylfunicone is consistent with previous reports where polyketides suppressed oomycete growth.^42^ Although the exact cellular mechanism by which 3-*O*-methylfunicone exerts its antifungal effect is not yet fully understood, elucidating its mode of action against pathogenic fungi will require more detailed studies.

## 4 Conclusions

This study demonstrated the effectiveness of a dereplication-based HRMS strategy in guiding the isolation of 3-*O*-methylfunicone, a bioactive polyketide produced by endophytic fungal co-cultures. The compound exhibited significant inhibitory activity against *P. palmivora*, one of the major causal agents of cocoa diseases. These results highlight the potential of 3-*O*-methylfunicone as a promising natural antifungal agent and reinforce the value of endophyte-derived metabolites in developing sustainable alternatives for crop disease management.

To advance its potential application in agriculture, further studies should focus on elucidating its cellular mode of action, assessing its antifungal spectrum, and evaluating its efficacy *in planta*. Additionally, exploring direct co-cultures of these endophytes with *P. palmivora* could provide deeper insights into pathogen-induced metabolite production and lead to the discovery of novel compounds with targeted antifungal properties.

## Supplementary Material



## Notes

Macroscopic morphology of 7-day cultures of the evaluated endophytic fungi, *Aspergillus pseudonomiae* J1 (A) and *Talaromyces pinophilus* J6 (B) (Figure S1); phylogenetic placement of *Aspergillus pseudonomiae* J1 (A) and *Talaromyces pinophilus* J6 (B) (Figure S2); annotated specialized metabolites and their distribution among *Aspergillus pseudonomiae* J1 and *Talaromyces pinophilus* J6 single and dual cultures (Table 1); HRESIMS spectra (positive ion mode) of phlorone (A), 4-hydroxy-2,5-dimethylcyclohexa-2,5-dien-1-one (B), isoboonein (C), coniferyl alcohol (D), and 3-*O*-methylfunicone (E) (Figure S3); 500 MHz ^1^H NMR spectrum of 3-*O*-methylfunicone registered in CD_3_OD (Figure S4); 125 MHz ^13^C spectrum of 3-*O*-methylfunicone registered in CD_3_OD (Figure S5); heteronuclear HSQC (500 MHz for ^1^H; 125 MHz for ^13^C) contour map of 3-*O*-methylfunicone registered in CD_3_OD (Figure S6); heteronuclear HMBC (500 MHz for ^1^H; 125 MHz for ^13^C) contour map of 3-*O*-methylfunicone registered in CD_3_OD (Figure S7); HRESIMS spectrum of 3-*O*-methylfunicone (positive ion mode) (Figure S8) (PDF)
