# Supplementary material for: Dereplication-Guided Isolation of 3‑O‑Methylfunicone from Endophytic Fungal Co-culture and Its Inhibitory Effect on Phytophthora palmivora
Source: ACS Omega. 2025 Nov 14;10(46):56587–96. doi: 10.1021/acsomega.5c08920 (PMC12658600; doi:10.1021/acsomega.5c08920)
Supplement: Supplementary file 1 [file ao5c08920_si_001.pdf]

Dereplication-guided isolation of 3-*O*-methylfunicone from endophytic fungal co-culture and its inhibitory effect on *Phytophthora palmivora*

Stéfane M. Q. Santos, [a,+] Marcus V. A. Marques, [a,+] Cecília L. S. Pereira, [a] Henrique B. da Silva, [b] Vinicius Palaretti, [c] Viviani N. Takahashi, [c] Sônia C. O. Melo, [b] and Eliane O. Silva [a,*]

[a] *Department of Organic Chemistry, Institute of Chemistry, Universidade Federal da Bahia, Salvador 40170-115, Bahia, Brazil*

[b] *Departament of Biological Sciences, Universidade Estadual de Santa Cruz, Ilhéus* 45662900, Bahia, Brazil

[c] *Department of Chemistry, Faculdade de Filosofia, Ciências e Letras de Ribeirão Preto, Ribeirão Preto 14040900, São Paulo, Brazil*

+ these authors contributed equally to this work

*Corresponding author: elianeos@ufba.br

Stéfane M. Q. Santos: 0000-0003-3764-2093

Marcus V. A. Marques: 0009-0002-5594-2954

Cecília L. S. Pereira: 0009-0009-9355-8196

Henrique B. da Silva: 0000-0001-8422-8982

Vinicius Palaretti: 0000-0002-1937-8834

Viviani N. Takahashi: 0000-0002-5092-3423

Sônia C. O. Melo: 0000-0002-7252-137X

Eliane O. Silva: 0000-0001-9121-0481

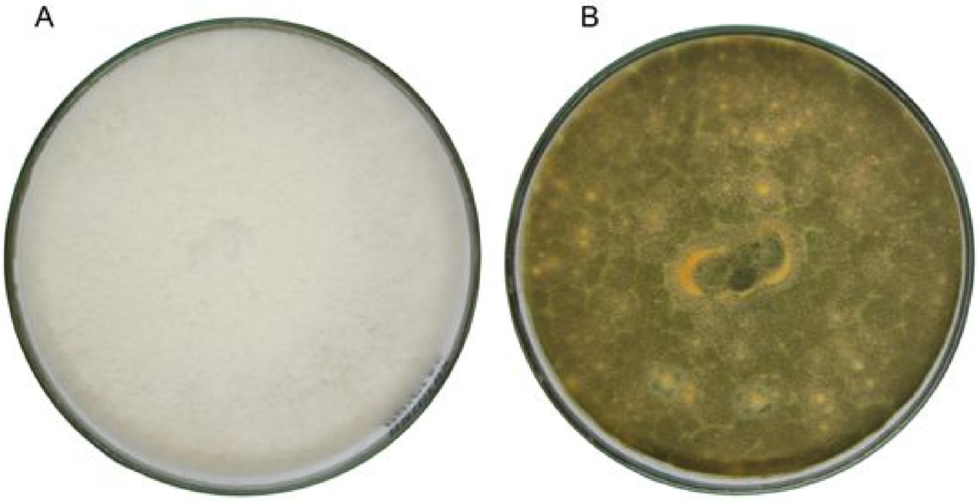

Figure S1. Macroscopic morphology of 7-day cultures of the evaluated endophytic fungi, *Aspergillus pseudonomiae* J1 (**A**) and *Talaromyces pinophilus* J6 (**B**)

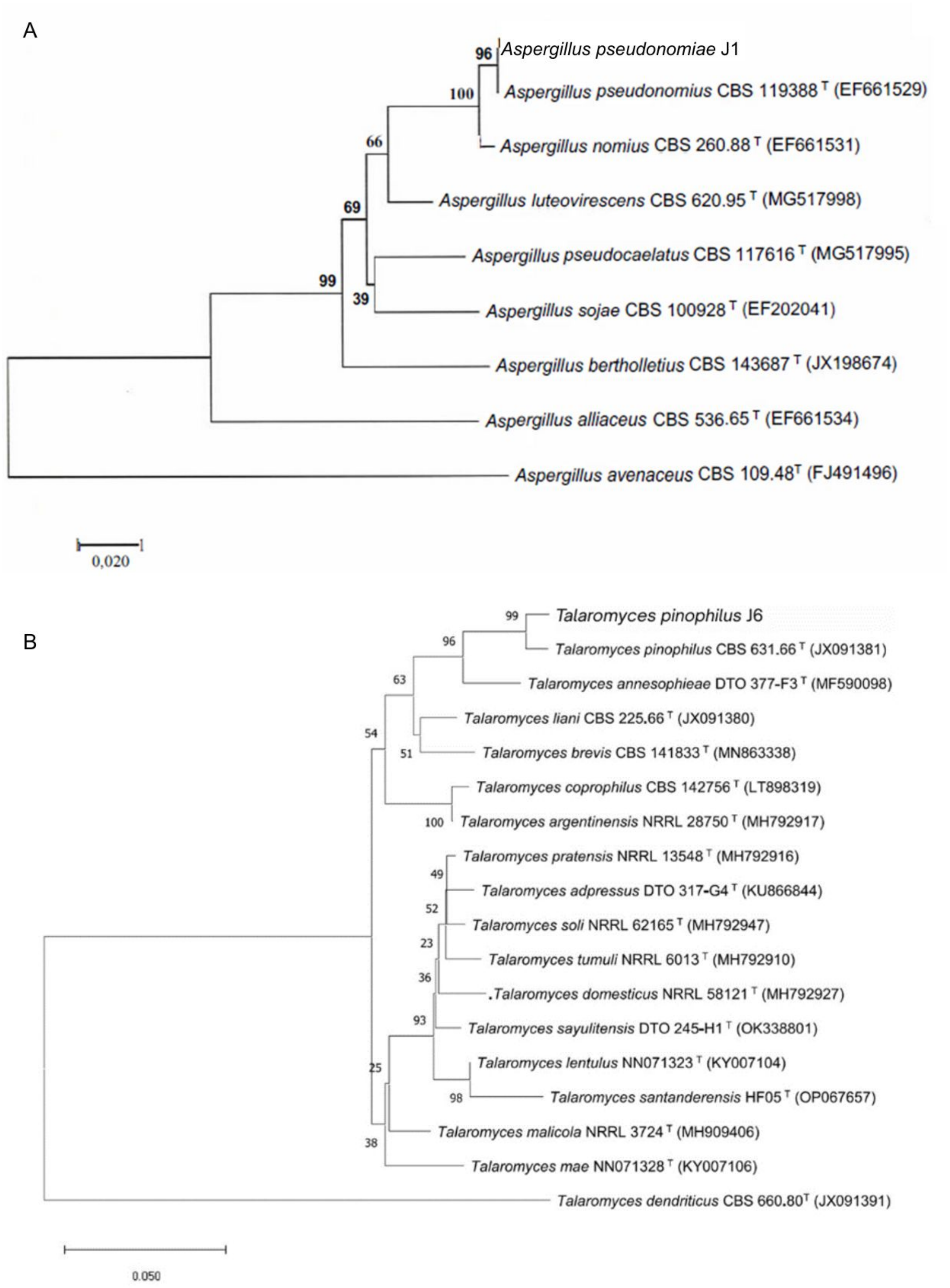

Figure S2. Phylogenetic placement of *Aspergillus pseudonomiae* J1 (**A**) and *Talaromyces pinophilus* J6 (**B**), based on calmodulin and β-tubulin gene sequences, respectively. The DNA sequences of the J1 and J6 strains were compared with reference sequences deposited in GenBank. Phylogenetic trees were constructed using the Neighbor-Joining

method in MEGA 6. Bootstrap analysis was performed with 1,000 replicates, and support values are indicated at the nodes. Scale bars represent 0.020 (**A**) and 0.050 (**B**) substitutions per nucleotide site.

Table S1. Annotated specialized metabolites and their distribution among *Aspergillus pseudonomiae* J1 and *Talaromyces pinophilus* J6 single and dual cultures. All data were exported from MSDial software

Alignment ID	Average Rt(min)	Average m/z	Metabolite name	Adduct type	J1_single culture	J6_single culture	Dual culture
7	3.954	102.12856	Unknown		895333772	440091822	808591342
9	3.601	104.10760	Unknown		167068208	869312842	124129101
10	4.024	104.96387	Unknown		846124863	888273978	293548513
11	15.450	104.96399	Unknown		350067320	61974955	200679950
12	2.994	105.07042	Unknown		518085036	238585047	196946001
14	18.320	107.08642	Unknown		121628868	133103051	528812831
16	14.937	109.06539	Unknown		14443543367	328889777	6724628882
17	15.827	109.06567	Unknown		12671422372	93771800	796620658
22	4.182	110.06048	Unknown		2687482900	631305171	1148181261
23	7.614	110.06064	Unknown		2073947096	87767413	688755129
27	16.209	111.08125	Unknown		9001948720	514410905	10929878287
41	3.711	118.08667	Unknown		166655209	5571715285	233673197
54	15.465	125.09681	Unknown		486446327	358493753	1038222135
55	3.896	126.05536	Unknown		1023740294	318341189	491735030
56	14.009	127.03947	Unknown		2106589197	432459765	1391296056
75	11.716	133.07678	Unknown		148061047	1107939872	1059303009
77	24.917	135.11736	Unknown		938804980	1090500060	751993143
79	14.951	137.06007	phlorone	[M+H]+	35947203400	1063560915	21238521692
82	18.424	137.06024	Unknown		1990940516	490223275	2247165755
88	15.895	139.07600	4-hydroxy-2.5-dimethylcyclohexa-2.5-dien-1-one	[M+H]+	11208854689	241327032	12872422123
102	20.849	143.07086	Unknown		2641981958	2449112281	2578335391
121	24.357	153.12790	Unknown		1789981150	162777479	272678276
122	14.937	155.07065	Unknown		23930751692	514488295	13943590982
130	16.035	157.08672	Unknown		1991658292	792620386	2555959282
132	24.581	158.15448	Unknown		410335898	395047203	402400134
147	17.500	165.08887	Unknown		1019061590	716393697	744644026
149	3.847	166.08659	Unknown		675231765	120391620	510218115
151	21.361	167.07086	Unknown		1006027825	622807353	391862783
155	17.023	169.05002	Unknown		8618991276	768340135	1404601990

157	13.612	169.08681	Unknown		390335149	372921169	381844437
161	18.103	171.10242	isoboonein	[M+H]+	1131541709	1343515559	10210968015
165	25.226	171.13866	Unknown		1372610270	1373689787	1628156221
166	26.302	171.13872	Unknown		900686160	270386266	1451126226
169	19.574	173.11816	Unknown		303262290	297403958	2454888240
170	15.873	174.11308	Unknown		1479144674	90233466	1553518446
171	18.849	175.09708	Unknown		1082489882	1139731441	1017142520
172	22.000	175.09712	Unknown		706744805	724635067	285853789
175	22.497	175.09718	Unknown		706744805	724635067	584340280
176	20.971	175.09724	Unknown		2556229480	2627072570	2365994491
177	24.005	175.14902	Unknown		456571601	77132927	422385712
180	14.951	177.05267	Unknown		7912527972	505406762	6108181464
182	12.919	179.06841	Unknown		233519762	259410645	288625848
183	15.965	179.06868	Unknown		3332621316	348058308	4990031754
187	19.327	181.08420	Unknown		1152113981	1147691937	1167086874
188	23.248	181.08635	coniferyl alcohol	[M+H]+	7084203020	623699197	2982151507
191	28.244	181.15935	Unknown		589213299	468759275	377397498
192	28.623	181.15947	Unknown		194900012	450777433	631836545
193	18.778	183.06377	Unknown		2405567372	2044449562	2059069057
196	3.917	183.08684	Unknown		469081573	1905809120	288548152
203	26.693	185.15427	Unknown		685633199	677212485	368463127
204	28.485	185.15446	Unknown		222998802	206259156	676446647
205	27.125	185.15451	Unknown		680785036	665974307	678580811
206	15.705	187.09737	Unknown		119452572	288136183	603213607
208	23.773	187.14883	Unknown		593512930	595226377	399176728
212	13.835	189.11317	Unknown		18485307	29692668	208848554
214	15.860	190.10815	Unknown		177118436	489117418	171520157
219	14.225	192.06639	Unknown		182702085	336318440	190644938
220	23.868	192.13907	Unknown		556226727	586050354	565211605
221	24.085	193.05025	Unknown		247595664	604679116	568521594
222	14.645	193.07150	Unknown		231194497	180873236	270590520
223	17.963	193.08458	Unknown		74842253	441492589	4431763483
224	25.069	193.12079	Unknown		1850845456	2027473236	2020709928
225	20.974	194.11859	Unknown		936994166	3992768282	2501067143
226	22.557	194.11870	Unknown		946528869	441048294	2730963055
228	19.905	195.10020	Unknown		1328061346	208536764	3109697659
229	18.954	195.10042	Unknown		1688176681	802991811	1667209280
233	20.310	197.08020	Unknown		879696048	1093979643	1669647427
234	20.012	197.08119	Unknown		6330677629	535787523	526778006
235	19.344	197.08153	Unknown		2613396197	423802699	1799719959
237	22.441	197.11774	Unknown		2387666420	2060243378	2157287581
239	14.591	199.05841	Unknown		625347630	556602118	566349328
246	25.156	201.14946	Unknown		439306512	485259075	554254611
251	21.737	203.12852	Unknown		719792751	136217251	138281651
252	24.281	203.12866	Unknown		173604842	1824254425	1859242607

253	26.874	203.14380	Unknown		287287989	195266352	191310149
254	20.903	205.08441	Unknown		1538897414	1786094301	1591145664
258	22.949	207.13615	Unknown		325952986	511169820	474094600
260	26.666	207.13644	Unknown		1125740538	1059191826	383723406
262	27.092	207.13655	Unknown		1125740538	1059191826	1171574868
263	18.838	208.09798	Unknown		1756491760	2024371477	2673501611
264	18.661	209.07951	Unknown		2220234709	3150573176	3772214765
265	22.175	209.11552	Unknown		1772125871	853488200	1186108115
268	24.086	209.11584	Unknown		1918493972	1137599908	1454110704
269	22.628	209.11595	Unknown		1326290305	2086002673	2151508495
270	25.872	209.15204	Unknown		1394000646	1975276989	1773228941
271	27.165	209.15466	Unknown		612928470	526600098	564027715
272	16.687	211.05838	Unknown		2818177839	1996127751	1892948305
273	21.999	211.09479	Unknown		4122599903	1784046825	1843244528
274	22.707	211.09483	Unknown		1345488427	4493901532	4216376696
275	25.556	211.13129	Unknown		3929482822	4167776109	3863131768
277	15.028	211.14490	Unknown		1271197305	764650833	495967958
278	18.900	211.14513	Unknown		3376568833	2957683917	3931711768
287	26.128	217.14439	Unknown		445105411	1374472431	1056525201
289	29.444	217.15965	Unknown		315089623	70043195	335929396
290	20.282	219.06343	Unknown		1357468438	343605944	316234438
292	28.244	221.15198	Unknown		1035843268	978658158	623557001
294	27.393	221.15228	Unknown		602882547	777522712	810139492
295	3.885	222.07661	Unknown		130530813	362464807	103978581
296	22.577	222.11353	Unknown		109095764	602094936	2080693618
297	23.934	223.06108	Unknown		2214892389	1157439136	2034136903
298	23.354	223.06125	Unknown		235151259	160709221	1275495624
303	26.894	223.13136	Unknown		1114928513	1454939853	1130131650
306	18.303	225.07425	Unknown		2518298957	2773006216	2262671873
307	23.719	225.11035	Unknown		3870112223	1576126702	1631826269
308	24.471	225.11058	Unknown		457294191	3808752438	4061299176
310	20.471	225.11086	Unknown		1695647933	2377558978	2076448735
311	27.555	225.14700	Unknown		553320116	2692005338	2889234766
322	13.500	231.08704	Unknown		160499825	290043059	169417276
323	28.790	231.13902	Unknown		245415493	53880607	835841749
324	27.192	231.15976	Unknown		619048218	204516941	269599303
325	27.757	231.16000	Unknown		581861859	650726107	506273151
327	20.915	233.08191	Unknown		216886337	223341987	1240837156
330	9.961	234.11366	Unknown		266426638	243503434	321581276
333	21.170	235.09755	Unknown		571979821	458625713	983949945
334	29.081	235.16811	Unknown		442721540	427309181	319078010
335	23.122	235.17007	Unknown		229062514	687054254	616752930
336	9.627	235.17039	Unknown		130934727	79068110	322022925
337	19.224	236.11375	Unknown		1814307277	1942260985	2030310451
338	21.843	237.11044	Unknown		2176736138	447820688	539834568

339	22.826	237.11046	Unknown		2176736138	2528050533	2632026348
340	27.037	237.14700	Unknown		770441023	1436366832	1414377100
341	25.899	237.14703	Unknown		838031118	980811233	824997523
342	26.391	237.14725	Unknown		1285396298	867561510	986166648
344	25.452	239.12604	Unknown		2615480270	1233096655	1260641037
345	26.222	239.12628	Unknown		1538934918	1847579405	2961353336
348	14.612	239.14980	Unknown		84872535	176982707	94511309
358	25.211	245.11572	Unknown		172294123	420027082	329027606
362	28.894	245.17549	Unknown		243675070	69159335	95864191
365	12.668	248.14993	Unknown		308967423	95580053	114512067
373	14.931	251.07611	Unknown		212472981	30162478	219130273
374	20.928	251.09254	Unknown		177574046	268847161	1301848032
377	29.316	251.16264	Unknown		1153516087	1241833910	1016667923
380	27.110	253.14182	Unknown		1620474110	1013129813	1055227282
382	27.730	253.14200	Unknown		1125913309	1730241751	1742615608
385	15.874	254.09984	Unknown		213456589	44625280	396266172
386	15.114	254.99611	Unknown		386166587	76030292	358669653
387	14.728	254.99615	Unknown		238498720	76030292	301624913
397	4.097	258.11069	Unknown		400232429	492209965	536075543
403	15.282	261.12466	Unknown		273724841	122460600	249247577
404	14.108	261.13156	Unknown		182300083	136931800	165071363
405	16.035	262.08600	Unknown		137483393	62712222	212908429
419	10.602	268.10486	Unknown		324552850	213829112	205649164
420	14.831	269.01208	Unknown		43490472	25452622	167912778
422	15.873	269.10522	Unknown		154577956	31485327	154104761
432	12.001	274.07190	Unknown		24320437	231871995	202964199
437	18.573	277.10568	Unknown		166455092	229711283	87144337
460	27.340	288.29059	Unknown		362598933	103238407	77840304
459	28.379	288.29059	Unknown		1331777316	853657748	776154782
461	27.678	288.29105	Unknown		125146902	351344562	324454091
466	22.317	291.08777	Unknown		32903300	482609475	699219679
474	25.768	295.19131	Unknown		1773164579	53054442	314886325
481	15.312	300.20251	Unknown		1654391048	1239178933	639325663
483	3.270	301.14154	Unknown		291949715	137261012	255584767
484	18.397	301.14203	Unknown		2464418689	779000947	2113242309
486	14.897	301.14236	Unknown		664033546	455188021	1054644183
520	3.270	317.11566	Unknown		55538254	305616358	57629121
531	15.460	320.11868	Unknown		238022408	11191288	137237773
542	14.911	328.10751	Unknown		1250558658	11473115	729393774
544	5.295	329.32465	Unknown		2255369955	2563213005	1399501838
545	3.527	329.32489	Unknown		1219683009	1265360743	1572551353
547	28.380	331.28534	Unknown		218827681	1766488490	1438030702
549	24.744	332.06033	Unknown		218110689	88158927	846265671
553	26.719	333.16833	Unknown		192665867	1824160908	1282319320
555	15.874	335.14801	Unknown		370036817	7941094	603603881

559	29.612	335.22031	Unknown		540156421	60716981	376740328
575	14.911	347.08102	Unknown		347024654	13756147	249866572
576	26.092	348.31241	Unknown		207531289	440685490	147064481
578	29.444	349.19989	Unknown		264724753	132329653	423390655
586	15.916	355.08578	Unknown		43282527	4215125	49103755
593	5.279	357.35590	Unknown		256450436	488742206	182688803
601	4.484	360.15057	Unknown		2964883	78905266	122430680
605	14.911	363.05334	Unknown		423780681	3035411	295584757
610	16.116	367.08551	Unknown		143296717	54118341	296625000
624	24.553	375.10904	Unknown		82521820	2127937547	1161073323
627	24.962	376.28610	Unknown		87762062	553926511	250015322
635	14.992	381.06345	Unknown		28560459	3780734	29876605
640	3.865	383.09601	Unknown		595270	53737787	2448521
645	28.893	385.20212	Unknown		1390518322	125728073	171896840
650	15.177	388.17639	Unknown		60847866	1041865	34692682
655	23.956	389.12439	3-*O*-methylfunicone	[M+H]+	42279702	16855434756	13042678531
658	26.599	391.16431	Unknown		136705781	37058181	207631855
661	14.911	393.08728	Unknown		120724184	4522096	111151533
666	24.540	397.09097	Unknown		180499882	3452085648	2186861230
668	15.852	397.11868	Unknown		56960738	184941	128639308
671	3.905	399.05917	Unknown		2198222	99642788	5174086
682	29.246	407.18423	Unknown		1045975684	6286402	181575624
683	29.724	407.18451	Unknown		235974723	41304837	1725918077
685	23.936	408.09802	Unknown		28290419	336766860	215209708
686	14.971	409.05969	Unknown		106360565	352135	160878895
692	24.188	411.10629	Unknown		127270662	15479934330	13455227805
697	24.308	413.11197	Unknown		117086421	650912083	645509234
698	26.411	413.21652	Unknown		160056477	83992414	676874675
699	29.564	413.23346	Unknown		2538356135	75631592	603456504
704	26.314	415.23816	Unknown		224444657	718188338	286279763
716	24.506	423.17917	Unknown		290207335	17001514	226864128
717	27.136	423.17953	Unknown		139133431	60332888	260170851
733	29.504	431.24423	Unknown		387347839	22735992	136041507
736	23.999	433.07172	Unknown		85520079	6505596	550100742
740	24.145	435.06729	Unknown		74141722	90708916	223991107
742	29.275	435.29706	Unknown		435206816	186518345	611589041
745	27.031	439.17407	Unknown		185292335	69692179	169032468
746	28.231	439.17416	Unknown		428034087	54616790	245765833
747	24.747	439.17432	Unknown		107876297	5800997	74362599
748	23.483	439.17462	Unknown		139022728	7567867	181271259
749	22.861	441.18982	Unknown		316520440	2444485	108810958
750	22.962	441.19049	Unknown		183010283	34081557	332862126
751	24.866	441.19058	Unknown		311836190	17391074	493564797
752	21.280	441.19138	Unknown		159966654	25976909	99394951
772	29.763	451.21082	Unknown		359351175	41117734	1289706442

775	29.369	452.32327	Unknown		6635453545	574753609	1990510258
777	27.659	453.22620	Unknown		1413973215	77008037	1302814037
778	25.328	453.22665	Unknown		142094182	19245282	215752927
779	29.423	454.22940	Unknown		1953550116	82867996	596817837
780	23.999	455.05380	Unknown		294783714	4203779	522534270
781	24.647	455.05399	Unknown		179022748	2657828	221698528
782	23.483	455.16931	Unknown		294514986	4054726	191485280
784	25.428	455.24203	Unknown		398614519	5117721	466288736
785	21.423	455.24243	Unknown		203981720	24525333	229471743
786	25.227	457.24466	Unknown		443254459	5513443	187594197
787	27.843	457.25806	Unknown		695747403	5199496	1024599266
788	29.261	457.27866	Unknown		3704301138	1731869267	4889665489
791	23.082	459.20065	Unknown		325931647	24874591	141616592
794	25.869	460.23401	Unknown		324657920	5901990	131910130
796	26.168	461.21866	Unknown		112899161	1927159	180051780
797	26.971	461.21869	Unknown		112899161	2891166	90293872
798	24.081	462.25021	Unknown		389389621	327224750	309764315
799	20.593	462.25073	Unknown		97060742	3074007	78260976
800	24.922	462.25079	Unknown		389389621	6519404	953809677
801	22.630	462.25085	Unknown		58280949	36181497	124208713
803	25.628	463.23438	Unknown		412640675	1866948	284632076
804	24.583	464.19748	Unknown		89053962	33242567	402401092
809	14.911	465.37744	Unknown		22055015	71500	27668989
810	24.185	467.20566	Unknown		739696627	8874302	120489092
811	25.047	467.20590	Unknown		318070467	15925037	3422174807
812	26.088	467.20657	Unknown		300314312	65396132	673651657
814	20.308	468.29745	Unknown		75131007	50087999	52969207
815	21.820	469.18497	Unknown		120914372	971036	72794694
816	23.945	469.22101	Unknown		235549612	225139	165781140
817	25.728	469.22113	Unknown		1905092062	1608222	638292293
818	29.663	469.22122	Unknown		506920234	5221605	344425926
820	20.981	469.22131	Unknown		125974682	33067533	108134906
819	27.906	469.22131	Unknown		542138448	31868439	232147438
829	29.038	473.25327	Unknown		102681000	17023558	163865183
832	29.159	475.20798	Unknown		199337136	2337040	47558054
835	18.615	476.30826	Unknown		135596865	97973029	117523585
837	23.894	479.22681	Unknown		558583955	65932433	87262533
838	24.826	479.22702	Unknown		1064726068	953721	194497808
840	19.730	479.28018	Unknown		64280163	66349754	82685943
842	24.667	483.20044	Unknown		277241828	3997065	190520971
843	25.949	483.20047	Unknown		1110556768	60418657	715581272
844	23.140	483.20053	Unknown		222890397	3016588	128500618
845	20.822	483.20059	Unknown		147410510	72049764	86142909
846	28.649	483.23691	Unknown		254457013	36780245	110772121
848	23.785	485.21652	Unknown		471329189	3451724	144799064

849	25.629	485.21692	Unknown		1187741658	10942280	1228974269
850	27.482	485.21692	Unknown		102604500	7930262	123642520
851	22.520	485.21713	Unknown		680262273	10564358	768456732
852	20.754	485.28796	Unknown		145590903	97033218	114131562
864	20.992	498.31274	Unknown		72077828	32921833	64062878
865	23.231	499.19492	Unknown		185416733	15603816	68380199
866	27.833	499.19510	Unknown		222191870	91686148	91472623
867	21.183	499.19550	Unknown		118656242	85829673	121185237
868	24.306	499.19608	Unknown		195364774	3827013	178149544
869	29.520	499.36227	Unknown		14519709	210179902	120248852
870	24.847	501.21127	Unknown		427833874	29369211	226898309
879	29.804	510.11813	Unknown		211825639	4656561	55984764
881	20.889	513.32153	Unknown		34343800	21408432	23928667
882	26.289	513.34833	Unknown		89707232	58030039	101022400
884	29.091	515.26447	Unknown		161309371	310971330	89773586
887	14.911	517.11700	Unknown		291975459	76730	150851496
892	19.247	519.22266	Unknown		18208208	21469555	20674514
893	18.873	520.33423	Unknown		135733286	106655750	114854402
894	19.864	521.29840	Unknown		120316038	127631799	108555916
896	20.825	523.16022	Unknown		14793820	16588521	32252029
897	24.970	523.19354	Unknown		43221209	15955878	21706248
898	19.931	523.30664	Unknown		126967129	143394809	154535110
901	21.038	525.31256	Unknown		27533524	25509490	35869848
902	24.045	527.22736	Unknown		24786087	907433	23318549
903	26.517	527.22760	Unknown		50011151	34550276	46224307
905	3.865	529.15991	Unknown		80598	12352941	46207
906	24.647	529.17682	Unknown		63407840	7184332	123430946
915	25.388	541.40961	Unknown		26129221	58496678	28329644
918	19.985	543.31183	Unknown		145979267	164905033	143936834
919	21.092	543.99054	Unknown		24599671	27029613	27688073
920	24.893	545.14923	Unknown		28801253	15735729	52333692
935	19.091	564.36096	Unknown		102935898	92179667	88501489
936	22.380	565.17114	Unknown		16284749	5194166	35222057
937	20.079	565.32440	Unknown		198486696	216673498	189332383
938	20.133	567.30157	Unknown		208918709	229085319	256077225
942	20.174	587.33807	Unknown		225302514	252132801	217068653
943	20.242	590.31799	Unknown		130950192	217922991	154642832
945	24.600	602.15686	Unknown		28153320	85577601	46224111
949	20.283	609.35101	Unknown		271200065	281033038	236795103
951	20.337	612.33081	Unknown		153102524	242401489	171494029
958	28.754	641.44135	Unknown		50545825	13212936	94694159
959	29.117	642.31482	Unknown		33069639	16888598	32884064
963	20.458	653.37750	Unknown		276015583	287269062	230120578
964	20.525	656.35651	Unknown		226447969	249853592	253153836
966	28.350	665.38654	Unknown		45182513	3000643354	96806693

971	27.549	683.54584	Unknown		46825521	2584424349	461178780
974	3.865	689.27277	Unknown		53990	17024480	59629
976	20.660	697.40399	Unknown		177683074	201557086	177736367
985	20.808	741.43060	Unknown		72043810	95059545	95622424
986	3.865	742.18402	Unknown		160943	8565975	76298
989	29.484	765.49438	Unknown		13415134	219021	60270519
991	23.915	769.21313	Unknown		125530	17474597	14383264
993	24.464	771.19281	Unknown		669550	42339312	24582185
994	23.354	771.19330	Unknown		1909230	116381	19686967
999	24.403	785.20801	Unknown		140012	24596268	19685708
1004	23.956	799.22375	Unknown		259446	1090693047	855015904
1011	29.744	827.40021	Unknown		83491272	128114	20513350
1012	24.787	833.17261	Unknown		501218	13522649	13382072
1014	29.424	837.42035	Unknown		81210741	275906	17002678
1021	26.153	911.42279	Unknown		13543964	742736	116964204

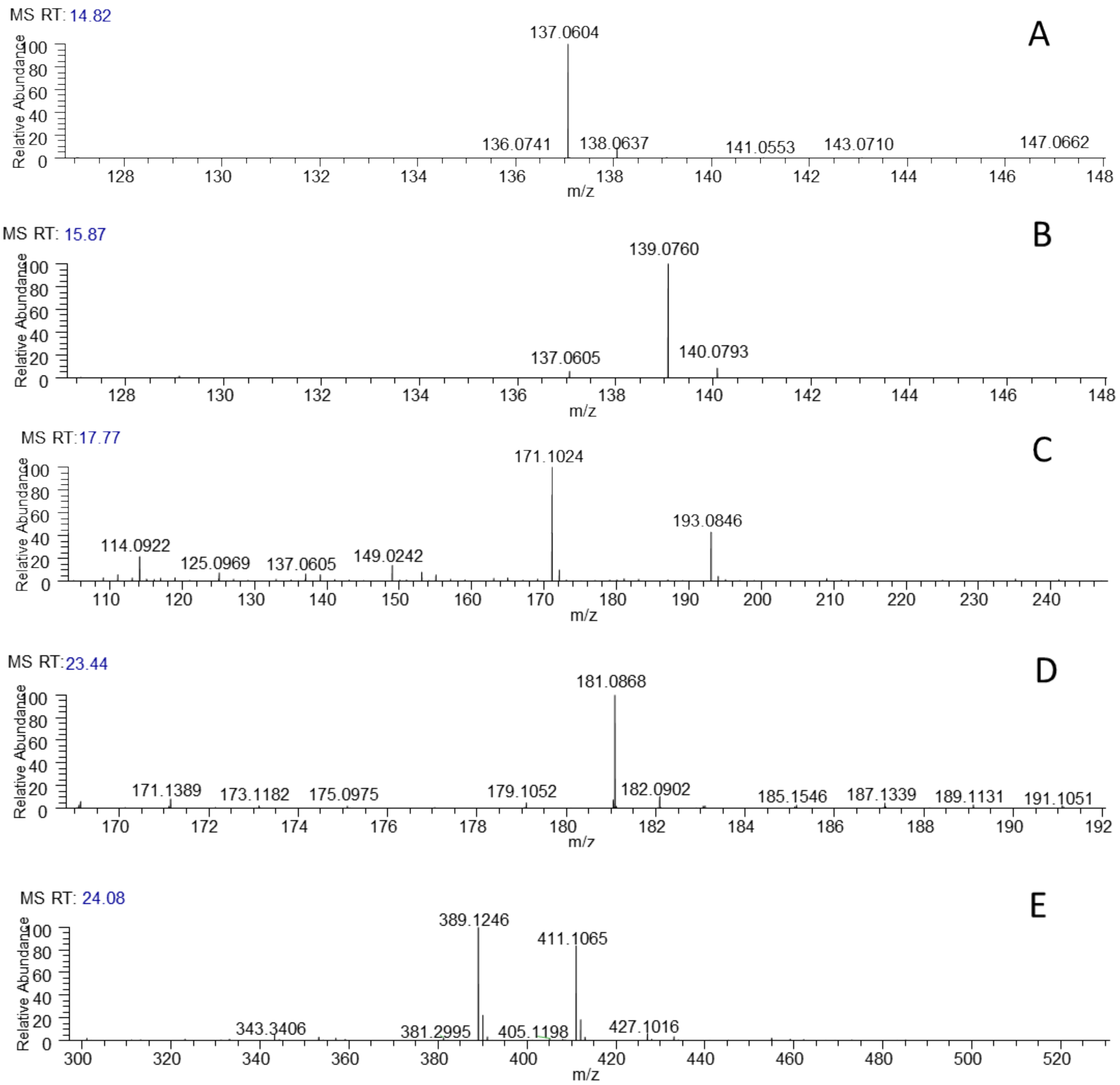

Figure S3. HRESIMS spectra (positive ion mode) of phlorone (**A**), 4-hydroxy-2,5-dimethylcyclohexa-2,5-dien-1-one (**B**), isoboonein (**C**), coniferyl alcohol (**D**), and 3-*O*-methylfunicone (**E**).

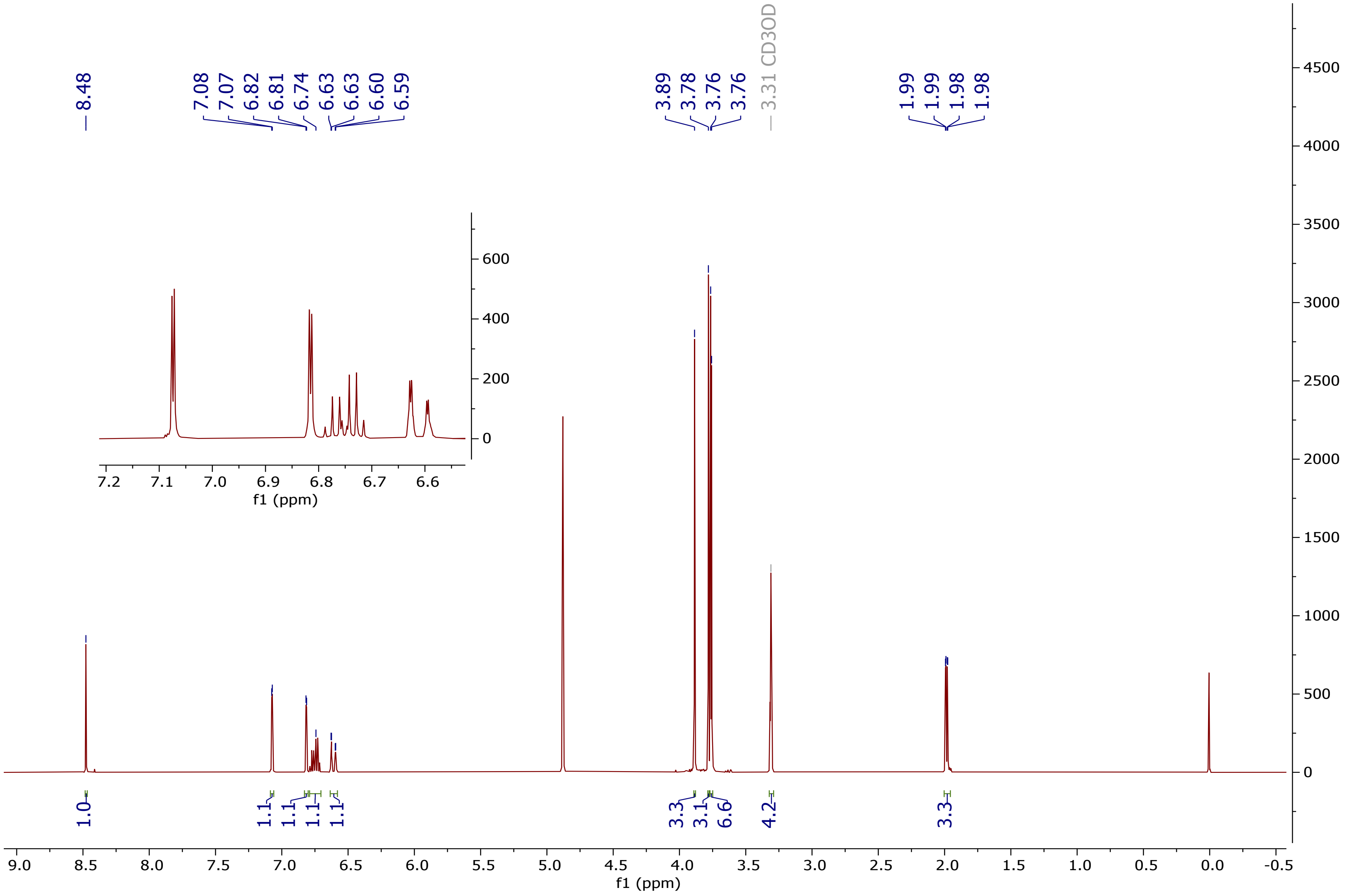

Figure S4. 500 MHz ^{1}H NMR spectrum of 3-*O*-methylfunicone registered in CD_3OD

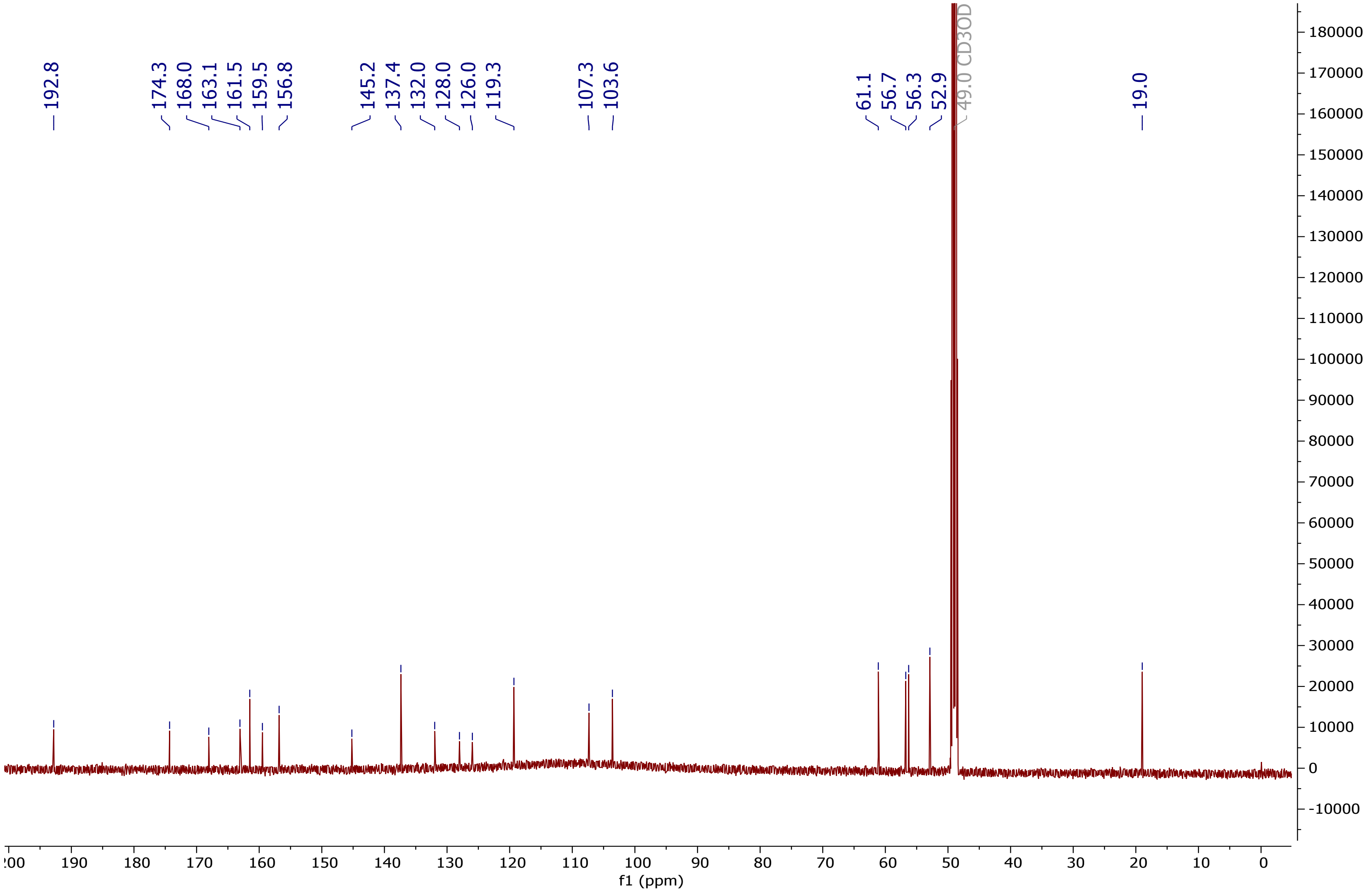

Figure S5. 125 MHz ^{13}C spectrum of 3-*O*-methylfunicone registered in CD_3OD

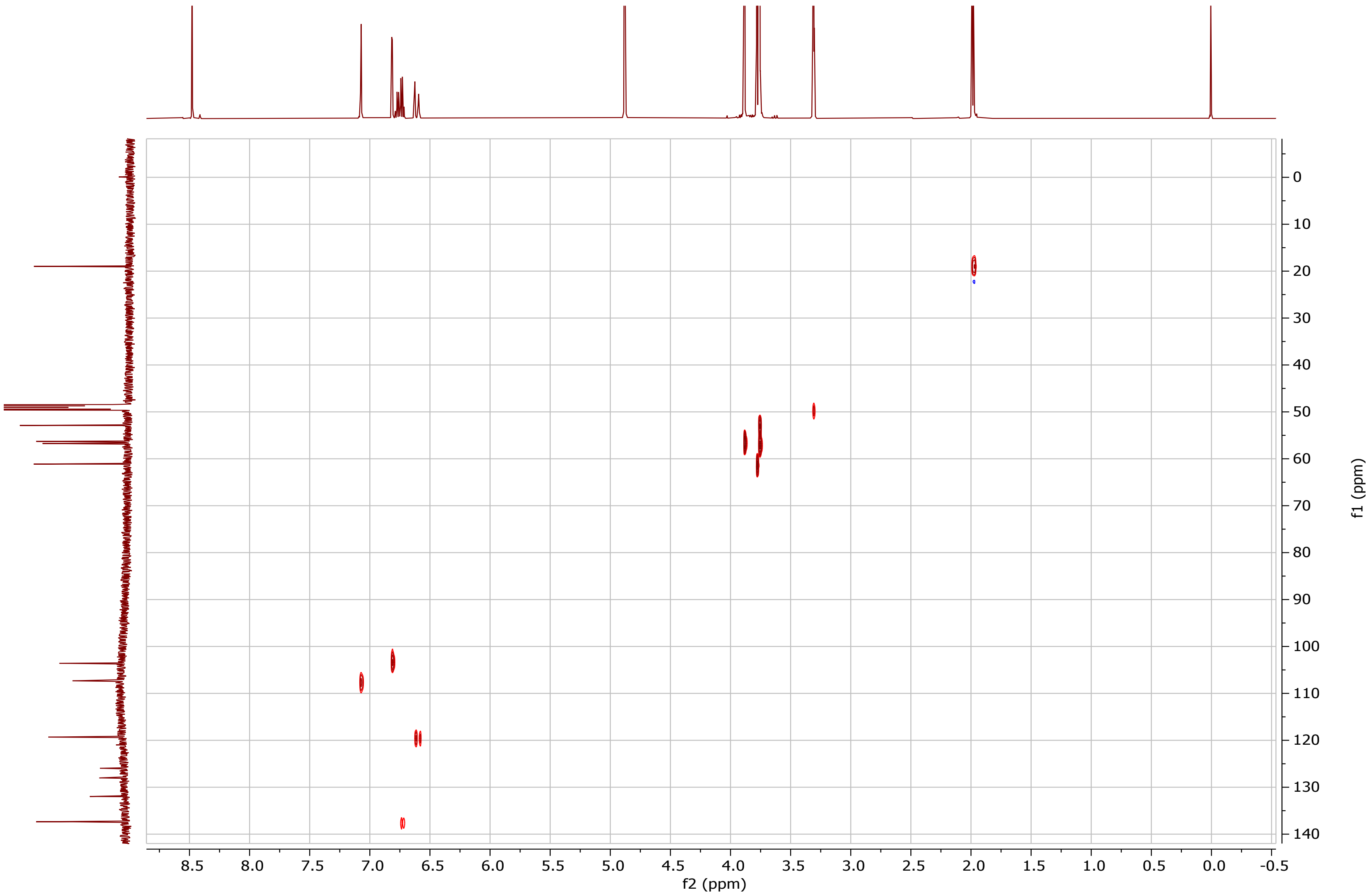

Figure S6. Heteronuclear HSQC (500 MHz for ^{1}H; 125 MHz for ^{13}C) contour map of 3-*O*-methylfunicone registered in CD_3OD

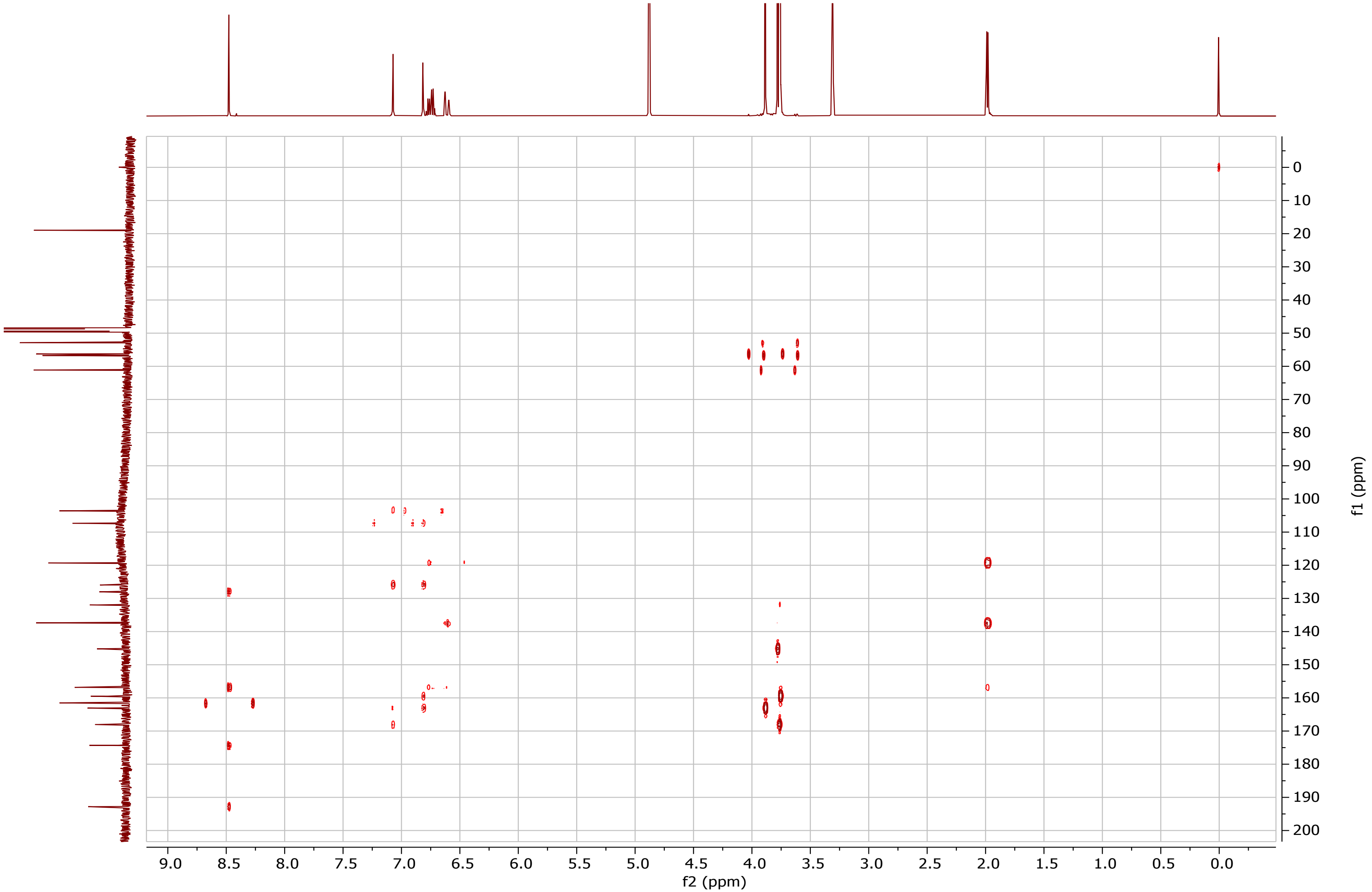

Figure S7. Heteronuclear HMBC (500 MHz for ^{1}H; 125 MHz for ^{13}C) contour map of 3-*O*-methylfunicone registered in CD_3OD

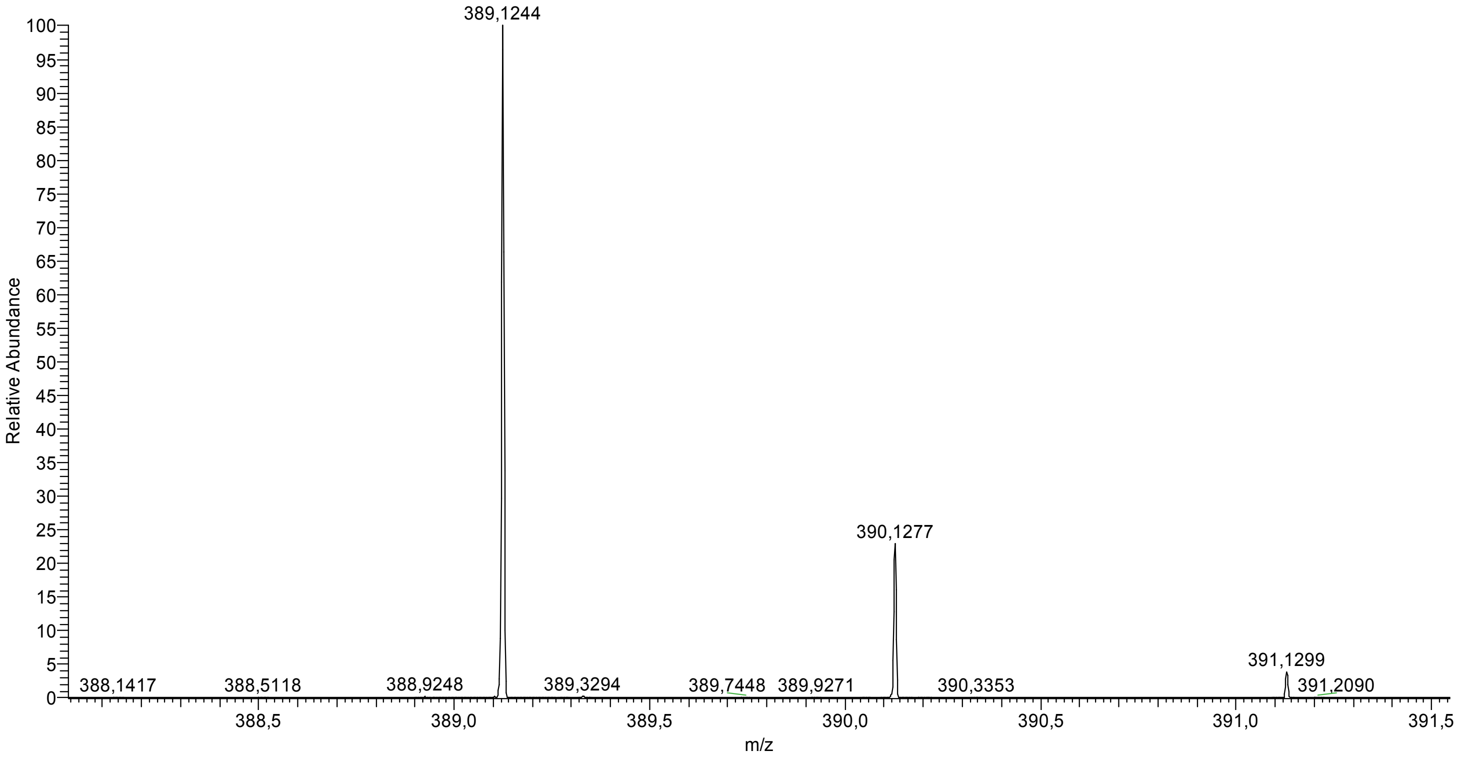

Figure S8. HRESIMS spectrum of 3-*O*-methylfunicone (positive ion mode)
